# Supplementary material for: Analysis of PPARγ Signaling Activity in Psoriasis
Source: Int J Mol Sci. 2021 Aug 10;22(16):8603. doi: 10.3390/ijms22168603 (PMC8395241; doi:10.3390/ijms22168603)
Supplement: Supplementary file 1 [file ijms-22-08603-s001.zip › Supplemental materials_Analysis of PPARg signaling activity in psoriasis/Pathway models/Models images and html files/PPARG inhibits phenotypes assotiated with psoriasis/142084.html]

aging


# Cell Process aging

|  |  |
| --- | --- |
| URN | urn:agi-gocellproc:0007568 |
| Total Entities | 2 |
| Connectivity | 6860 |
| Name | aging |

---

|  |  |
| --- | --- |
| ChildConcepts | senescence |
|  | skin aging |

---

|  |  |
| --- | --- |
| Pathway | Longevity Related Drugs |
|  | Genes with Mutations Associated with Aging in Humans |
|  | High Level of Homocystine Effects (Methylation Cycle) |
|  | Extracellular Matrix Reformation in Aging |
|  | TGF-beta Signaling in Skin Aging |
|  | POMC Activation in Keratinocytes |
|  | PPARG inhibits phenotypes assotiated with psoriasis |
|  | Neighbors of aging |
|  | Overview. Diseases of the musculoskeletal system (chapter 12) |
|  | Overview. Diseases of the nervous system (chapter 5) |
|  | Overview. Diseases of the eye (chapter 6) |
|  | Overview. Diseases of the ear (chapter 7) |

---

|  |  |
| --- | --- |
| MedScan ID | 4200450 |

---

|  |  |
| --- | --- |
| Alias | aging process |
|  | agings |
|  | aging processes |
|  | aging |
|  | age induced changes |
|  | ageing |

---

|  |  |
| --- | --- |
| GO ID | 0007568 |

---
